# Implementation of the integrated management of childhood illness with parasitological diagnosis of malaria in rural Ghana: health worker perceptions

**DOI:** 10.1186/s12936-015-0699-y

**Published:** 2015-04-23

**Authors:** Lawrence G Febir, Frank E Baiden, Justina Agula, Rupert K Delimini, Bright Akpalu, Mathilda Tivura, Nelson Amanfo, Daniel Chandramohan, Seth Owusu-Agyei, Jayne Webster

**Affiliations:** Kintampo Health Research Centre, PO Box 200, Kintampo, Ghana; Epidemiology Unit, Ensign College of Public Health, Kpong, Eastern Region Ghana; National Catholic Health Service, Project Fives Alive Christian Village KS 99, Kumasi, Ashanti Region Ghana; University of Health and Allied Sciences, Ho, Ghana; Disease Control Department, London School of Hygiene and Tropical Medicine, Keppel Street, London, WC1E 7HT UK

**Keywords:** Malaria, Perceptions, Integrated Management of Childhood Illness, Health workers

## Abstract

**Background:**

Timely and appropriate management of febrile illness among children under five years of age will contribute to achieving Millennium Development Goal-4. The revised World Health Organization-Global Malaria Programme’s policy on test-based management of malaria must integrate effectively into the Integrated Management of Childhood Illness (IMCI). This study reports on perceptions of health workers on the health system factors influencing effective delivery of test-based diagnosis of malaria with IMCI.

**Methods:**

A qualitative study was conducted among a range of health workers at different levels of the health system in the Brong Ahafo Region of Ghana. Interview transcripts were transferred into Nvivo 8 software for data management and analysis. A frame-work approach at two levels was used in the analysis, which included the processes required for implementation of test-based management of malaria and the health systems context.

**Results:**

Forty-nine in-depth interviews were conducted. The National Health Insurance Scheme (NHIS) was perceived to have led to an increase in health facility attendance, thereby increasing the workload of health workers. Workload was reported as the main reason that health workers were not able to complete all of the examinations included in the IMCI algorithm. The NHIS financing guidelines were seen to be determining diagnosis and treatment practices by health-care givers. Concern was expressed about the erratic supply of malaria rapid diagnostic test kits (RDTs), the quality of RDTs related to potential false negative results when clinical symptoms were consistent with malaria. IMCI was seen as important but practically impossible to fully implement due to workload.

**Conclusions:**

Implementation of the WHO-revised IMCI guideline is confronted with a myriad of health systems challenges. The perceptions of front-line health workers on the accuracy and need for RDTs together with the capacity of health systems to support implementation plays a crucial role. The NHIS financing guidelines of diagnostics and treatments are influencing clinical decision-making in this setting. Further study is needed to understand the impact of the NHIS on the feasibility of integrating test-based management for malaria into the IMCI guidelines.

## Background

The Integrated Management of Childhood Illness (IMCI) initiative was developed by the World Health Organization (WHO) with the aim of reducing childhood mortality, particularly for children under five years of age [1]. IMCI aims to integrate management of the common conditions that children present with at health facilities, to improve the quality of care for children and reduce severe morbidity and mortality. IMCI recommended that every fever presenting at a health facility be treated as malaria in malaria-endemic areas based on the fact that the health system is weak in malaria-endemic countries and the capacity to conduct parasitological tests to confirm malaria was very limited [2]. The WHO-Global Malaria Programme (WHO-GMP) revised guidelines in 2010 stating that regardless of the age of the patient and the endemicity of malaria, case management of malaria should be test based [3] and that IMCI guidelines needed to be revised in accordance with this new policy. The successful implementation of a universal rule of ‘test and treat’ may, however, be challenging. For example, for rapid diagnostic test (RDT)-based management of malaria to be accepted and sustained, caregiver-health worker interactions need to be understood and improved [4]. Lack of adherence to negative parasitological tests has been reported in studies in several settings [5,6] including a hospital in the Kintampo Municipality, and has become a focus for studies of test-based diagnosis of malaria [7]. This study reports the perceptions of health workers on the wider issues they face in integrating parasitological diagnosis into their working practices within the context of existing health systems.

## Methods

### Ethics

The study was approved by the Institutional Ethics Committee of Kintampo Health Research Centre, the Ethics Review Committee of Ghana Health Service, and the Ethics Committee of the London School of Hygiene and Tropical Medicine. Approvals were obtained from the District and Municipal health management teams and the health centre managers. Consent was sought from individual respondents; they were not coerced to provide information.

### Study site

The study was conducted in seven of the 27 districts in the Brong Ahafo Region of Ghana. Malaria transmission is high in this part of Ghana with an entomological inoculation rate (EIR) of 269 infective bites per person per year and an annual parasite prevalence of 50% among children under five years of age [8]. *Anopheles gambiae* and *Anopheles funestus* have been identified as the main vectors in this area [9]. The use of insecticide-treated bed nets (ITNs) among children under five years of age in the Brong Ahafo region is 30.3% [10].

The structure of health facilities in the area is typical of other districts in Ghana: they include a district hospital, health centres and community-based health planning and services (CHPS) compounds. District hospitals provide support to sub-districts and communities for referrals, emergencies and training. The health centres provide basic curative care, disease prevention and maternity services. The CHPS compounds engage in outreach programmes and provide basic curative care including first aid. In 2004 Ghana’s National Health Insurance Scheme (NHIS) was introduced to replace the out-of-pocket fee for services provided at the health facility with the aim of promoting increased access to health services and improving the quality of services provided to those needing them.

### Sampling and study procedures

The study was undertaken between June 2009 and December 2010. Health workers were purposively selected for the study to represent the perspectives of a range of cadres at different levels of the health system. Respondents included staff at the district health management teams (DHMTs), health centre in-charges, pharmacists, clinicians and drug dispensers. In-depth interviews (IDIs) were conducted in English by two trained research officers. An interview guide was used and themes included current practices in the case management of febrile children, and perceptions of the new parasitological diagnosis-based policy. All interviews were digitally recorded and transcribed verbatim. Digitally recorded audio transcripts were exchanged between transcribers and vetted to make sure the typed transcripts matched the audio transcripts. Interview transcripts were transferred into Nvivo 8 software for data management and analysis.

### Coding and analysis

Data was coded by four of the authors (LG, BA, NA, and JW). After familiarization with the data a framework approach [11] was taken at two levels in order to take a holistic view of diagnosis within the overall case management of febrile children and to situate this within the health system as a whole. The first level of the framework was composed of the system’s effectiveness processes [12] including: 1) malaria diagnosed and an anti-malarial is prescribed; 2) given artemisinin based combination therapy (ACT); 3) given the nationally recommended ACT; 4) carer is told how to give the ACT; and, 5) carer knows how to take the ACT on exiting the health facility. The second level of the framework then added the health system’s context and was composed of the six building blocks of the health system as defined by WHO [13]; these were: governance, financing, human resources, health information, products and technologies and service delivery. Themes were developed within these six health system building blocks and based on content analysis of the data. Mind maps were used to display and discuss the results. Anonymized quotes were used to describe emergent themes.

## Results

A total of 49 IDIs were conducted. Themes emerging on the integration of parasitological diagnosis of malaria into IMCI were broadly divided into two categories: 1) the health system’s context within which parasitological diagnosis needs to take place; and, 2) service delivery of malaria diagnosis.

### The health system’s context within which parasitological diagnosis needs to take place

Three major themes on the health system’s context emerged, which included the NHIS, financing and supply and quality of RDTs.

### The National Health Insurance Scheme (NHIS)

Although not asked specifically in the IDIs, the NHIS was mentioned frequently by the interviewees. A number of issues were raised relating to the NHIS, including workload and over-diagnosis of malaria for financial gain. From the perspective of the interviewees, the NHIS has led to an increase in health facility attendance, thereby increasing the workload of health workers. High workload was repeatedly cited as the main reason that health workers were not able to complete all of the examinations included in the IMCI algorithm, particularly in hospitals. It was also mentioned by respondents from the hospitals (although not as frequently) that the NHIS was a reason for not requesting laboratory tests.

*“Following the introduction of the NHIS, the patient inflow at the hospital is so much that the clinician finds it difficult to go through the IMCI processes or ask every patient to go for laboratory investigation*.” (Hospital in-charge 3)

It appears that free entitlement of review visits, one of the benefits of NHIS, is a major contributor to the increased workload.

*“Now with the concept of NHIS, the client has two weeks within which he or she can be reviewed once or twice.”* (Hospital clinician 4)

The health workers interviewed perceived that the NHIS has led to over-diagnosis of malaria by some health facilities for financial gains. This is because the major form of payment from the NHIS to health facilities is for the treatments that they have prescribed.

*“The more the patients, the more the money you get. You see people come for review and they [health workers] write malaria treatment for them.”* (DHMT 2 )

*“Due to the NHIS, people are diagnosed with 3 cases even if you suspect one case. For NHIS, they only pay according to their guidelines and hence this increases malaria cases.”* (DHMT 4)

### Financing

#### Diagnosis-dependent reimbursement of treatment costs and the NHIS

Several health workers reported that the NHIS only pays health facilities for the cost of drugs to treat malaria, where the patient has been tested for parasites and confirmed as positive. They perceive that where a patient was not tested, or was tested and was negative, the NHIS would not reimburse the cost of ACT given to the patient. This was even perceived to be the case where parasitological diagnosis was not possible, where there were no laboratory facilities or capacity to conduct malaria microscopy, and where RDTs were not available.

*“The NHIS will not reimburse a facility if the lab test doesn’t show as positive in diagnosing malaria. This is a challenge for places without lab facilities.”* (DHMT 3)

### Facility level financing of RDTs

Health workers in charge of both hospitals and health centres were concerned about late reimbursements by NHIS to their health facilities for funds used in buying RDTs. Until the health facilities pay for outstanding debts with the regional medical stores, supply of RDTs are curtailed.

*“If the health facilities are going to procure these test kits [RDTs], it is going to create a problem especially considering the fact that the NHIS do not pay the facilities on time. With the attitude, the regional medical stores will also be reluctant to give us the kits because we are unable to pay our previous debts. But if the management (National Malaria Control Programme) provides the kits free of charge, it will help.”* (Hospital in–charge 3)

### Supply and quality of RDTs

In addition to issues commonly associated with the procurement supply chain management (PSCM) system, respondents mentioned problems relating to stock-out of donor-funded RDTs and inadequate and inconsistent supplies of RDTs.

*“They will start a project nicely I don’t know where the problem is coming from. At the receiving end and you will be waiting to get twenty, then you are getting five, the five becomes two, the two becomes one, and the one becomes zero. And there is nobody who can give reasons. At the end you can blame anybody.”* (Hospital in-charge 2)

*“If I am to go buy …….. then I cannot vow that there will be a regular supply [of RDTs] because ……….I think it’s from Global Fund and any donor supported thing can run out………”* (DHMT 4)

Respondents mentioned stock-out of RDTs for significant periods and stated that stock-out of RDTs would lead to presumptive treatment and result in an increase in the proportion of patients diagnosed with malaria, thus, demonstrating a perceived over-diagnosis of malaria by clinical methods, together with a lack of differentiation in the facility level statistics for confirmed and unconfirmed malaria.

*“Now I don’t have the kits for February so it is likely that the rate of malaria will be high in that month because I will be diagnosing based on the signs and symptoms patients present.”* (Health centre in-charge 6)

Most of the respondents were concerned about the quality of RDTs and the mistrust of negative results when they felt that the clinical symptoms were consistent with malaria. In these circumstances all respondents said that malaria treatment would be given, because they felt that the test was incorrect or that the parasite was “hiding”, showing significant doubt in the quality of the tests.

*“…..as long as the result comes out to be positive, I am sure it is malaria and I go ahead and treat. But then the rapid test comes out negative I have my doubts when there are all symptomatic and examination findings pointing to malaria. You still go ahead and treat malaria because the rapid test kit cannot be specific.”* (Hospital clinician 6)

*“Why is it that sometimes if the malaria test kit proves negative and I give treatment for other illnesses the condition of the child worsens after few days, but the moment I give artesunate amodiaquine to that same malaria negative child, he or she fully recovers.”* (Health centre clinician 3 [responded spontaneously at end of interview]

There were some suggestions that it would be useful to compare the RDT results with microscopy (referred to as ‘lab tests’) but that this had not been done and that the lack of laboratory facilities and therefore ability to reach alternative diagnoses influenced over-diagnosis of malaria.

*“For us we have not looked at their quality because we do not have anything to compare with, if we have a lab we could do it and compare with some lab test.”* (DHMT 2)

*“You know the health centre we don’t have labs……… for the hospitals, they can do other tests and see that this person is with malaria or this sickness. But here be the case that we the health centres don’t have labs....we have used it [RDT] to prove negative but there not other tests to prove short it’s other illness but we still believe it is malaria, we can give*.” (Health centre in-charge 5)

Apart from looking for other methods with which to compare RDT results, care providers perceive some RDTs to be of inferior quality because of difficulties in getting the required amount of blood for testing.

*“I think that the malaria test kits that we are using are inferior because after pricking the patient, how to scoop the blood samples into the kits for testing is a problem.”* (Health centre in-charge 4)

*“Sometimes the accuracy of the medicine used for testing malaria will not be there. For instance, we are told that the lab technician takes only a small sample of blood for testing and there are situations where the parasite may not be present in blood sample taken even though the patients has malaria. In two days time the same patient will prove positive for malaria when he or she is tested. The policy can still hold but there should be exceptional cases (about 5%) especially when the clinician or prescribe is sure beyond all reasonable doubt that the patient has malaria. That wouldn’t cause any harm on the artesunate amodiaquine therapy*.” (Health centre in-charge 8)

### Service delivery of malaria diagnosis

Issues that were raised in relation to IMCI, including workload, severity of illness, cadre of health worker, and training of health workers. When asked specifically about their views on the IMCI assessment algorithms all health workers said that they were important and should be conducted. However, most also stated that it was usually practically impossible to implement these assessments due to the workload, as mentioned above.

*“At times it escapes us [IMCI] due to the workload.”* (Clinician 5)

*“In the urban area hospitals, the clinicians may not be able to fully implement all the IMCI requirements because of the many patients they have to attend to. Spending too much time on one patient may not be helpful looking at the many patients waiting at the OPD to be attended to.”* (Health centre clinician 4)

Because of the workload, clinical judgment is used to select the patients who they would prioritize for both urgent examination and a more complete IMCI assessment. Febrile cases are not amongst those considered as urgent, nor needing the attention of a doctor. In hospitals, where doctors are usually present, febrile cases are more likely to be seen by medical assistants because they are considered non-serious.

*“When a doctor has so many patients to attend to, he or she will not get adequate time to implement or follow these requirements of IMCI. He may be forced to carry out these requirements on children who are very sick and need urgent attention due to the heavy workload.”* (Hospital in-charge 4)

*“This can be attributed to workload where you have a one man station, most of the time, the clinician will have the serious cases referred to him especially those cases of RTA [Road Traffic Accident] and hernia. The rest can be shifted to their Medical Assistants to handle.”* (Hospital clinician 4)

Very few health workers have been trained in IMCI in the districts and for this reason are not able to carry out all IMCI assessments. This is in part due to lack of trainers at the district level. There is a perceived need for more training of trainers so that each district is able then to have the capacity to train its health workers.

*“There are so many health facilities here within the district and just a handful of clinicians are trained in IMCI. So that is the main reason [why IMCI is not implemented]. It is only when we go out on our supervisory visits that we talk to our staff. But even then, there is the need to train them on IMCI.”* (DHMT 6)

*“So I think the best approach is to train trainers in each district so that staff in each district could be trained to perform IMCI requirements. They are only four in the region [IMCI trainers] hence it is difficult to get them sometimes.”* (DHMT 6)

### Pre-policy change practices on parasitological diagnosis in hospitals

Only a small proportion of the patients who visit hospitals were referred to the laboratory for malaria tests. It was more common for patients to be diagnosed based on clinical judgment. The majority of patients visiting the hospital, particularly those with fever or chills would be diagnosed and treated for malaria. A major reason for this was the number of patients and the feasibility that they could all receive a laboratory test.

*“I can say one quarter of them have passed through the lab…. But most of our malaria cases depend on the signs and symptoms…..the numbers, you can’t push all of them to the lab….if you see that the symptoms are clear and you rule other conditions out, the first line of treatment for every child coming in or every patient coming into our facility the first line of treatment is always malaria.”* (Hospital in-charge 2)

*“And even where we have the facilities we look at the workload, that is the number of patient that come to hospital, if they are all asked to go to the lab. And even in our system you know how cumbersome it is when we go to hospital you are asked to go here, there and so many things. So it’s like most of the diagnosis are now being through experience. It’s like in Ghana if you are having fever, chills then its malaria.”* (Hospital pharmacist 1)

### Microscopy *versus* RDTs

Respondents were asked about their preferences for malaria microscopy *versus* RDTs. Most clinicians preferred microscopy to RDTs when it comes to testing for malaria parasites. Reasons for this preference included knowing the severity of the condition (parasite density), species confirmation, trusting the microscopy results when clinical diagnosis was not clear. Most respondents however were not able to disassociate the concept of malaria microscopy *versus* RDT with the context of availability or non-availability of other confirmatory tests as other tests that will lead to better understanding of the cause of a fever can be done using microscopes and other laboratory equipment.

*“At the hospitals, apart from using the test kit, the microscope can be used to detect whether the malaria is +, ++, +++ to know the severity of the condition.”* (Hospital pharmacist 4)

*“The kits we are using now are for Plasmodium falciparum but we are living in farming communities where patients can come with the other species of malaria which can easily be detected by the microscope than the malaria test kits.”* (Hospital clinician 4)

The absence of laboratories in the health centres allows no other alternative than the use of RDTs, but again this was strongly associated with the perception of not being able to confirm alternative diagnoses and therefore treating for malaria even if negative.

*“You know the health centre we don’t have labs. So for us, I think we can do it. But for the hospitals, they can do other tests and see that this person is with malaria or this sickness. But here be the case that we the health centres don’t have labs....we have used it [RDT] to prove negative but there not other tests to prove short it’s other illness but we still believe it is malaria, we can give [treatment].”* (Health centre in-charge 5)

*“I think it would be easier to do this when the labs are working. If I am in a situation where I can only do a rapid test and cannot do full blood count, I cannot do other labs to get to the root of the cause of the fever.”* (Hospital clinician 2)

The use of microscopes will require other technical personnel and inputs for accurate diagnosis*.* The absence of which may lead to poor or improper diagnosis.

*“It is good in practice but you need to get all the resources or else it will be poorly done e.g., lab technicians, laboratory materials e.g., microscope, etc.”* (DHMT 4)

### Process of parasitological diagnosis

Respondents were asked when during the process of diagnosis and management of malaria parasitological diagnosis should be conducted. The responses included preferences for this diagnosis to be pre-consultation, during consultation and post-consultation.

Pre-consultation: Some clinicians preferred that RDTs are done before patients arrive at the consulting room with their reasons including that this would be satisfying for the carer.

*“If all are tested before coming to the consulting room, it will be better because the mothers find the test satisfying even if it proves negative.”* (Health centre clinician 6)

*“I would like the test to be carried out at the OPD so that the careers will come to me with the results for the treatment to be given.”* (Health centre clinician 3)

During consultation: A quicker method for confirming the diagnosis of malaria will aid in providing treatment. The absence of this poses a challenge to both prescriber and patient. This will solve the problem of congestion in the laboratories.

*“So if there could be a quick means of testing malaria, then it will be very helpful whereby even in the consulting room the doctor can easily do the test on his own and then go on with the treatment. Either than that if its only laboratory………,then it becomes very difficult on the part of the prescriber as well as on the part of the patient.”* (Hospital pharmacist 1)

*“For the diagnosing and prescription to be done test kits that give fast results like the glucometer should be supplied and malaria test carried out in the consulting room rather than allowing every patient to the lab to avoid congestion.”* (Hospital pharmacist 4)

Post-consultation: Despite the fact that according to current policy all febrile patients should be tested for malaria parasites before being prescribed a treatment for malaria, many clinicians when questioned preferred to see the patient first in order to decide whether they required a malaria test. Some described it as a waste of both the carer’s time and of laboratory inputs required for the test.

*“It will depend on the history the carer will give and the physical examination that will be conducted by the clinician to confirm if the child has malaria or not. I will prefer that the child be brought to me for the physical examination to be carried out and the signs and symptoms to be identified before deciding on using the RDT or referring him to the lab for malaria test to be done.”* (Health centre clinician 8)

*“I would prefer that the child comes to see me first before going for the test because it’s not all fever that is malaria so if the test is conducted before consultation, it is a waste of time for carer and waste of the reagent [kit] used.”* (Hospital clinician 4)

Most clinicians express worry about the amount of time a patient has to spend at the health facility due to the number of patients waiting to be attended to and long hours of waiting for the results of their test.

*“The policy doesn’t allow free supply of malaria test kits so they will continue with routine treatment of malaria. Look at xxxxx health centre that treats over 300 patients with few staff. They have only one effective microscope….. here we are saying should be waiting for their malaria result, and even how long it takes to treat them all.”* (DHMT 4)

## Discussion

Implementation of the WHO revised IMCI guidelines faces a wide variety of challenges given the weak health systems in most developing counties [14]. The findings of this study suggest that the problem is heightened by beliefs and habits of front-line health staff in health facilities in developing countries that are used to presumptive treatment and perceive every fever to be malaria. It is worth noting that this study was carried out in a high-transmission setting and the results may differ from any carried out in a low-transmission setting where there is less belief that every fever is malaria. In health facilities, malaria RDT kits are intermittently unavailable or there is a communication gap between managers of health facilities and DHMTs who are responsible for their medical supplies. In hospital settings where microscopes are available and remain the gold standard of diagnosis and treatment, other co-morbids are easily detected making the prescription of approved ACT quite easy. This is the ideal situation and this is what the new IMCI guideline seeks to implement. Application of the IMCI guidelines is constrained by lack of training and retraining of medical staff in hospitals.

This study found perceived high workload to be a challenge leading to poor adherence to guidelines. Similar factors, such as cost of training health staff, duration of IMCI procedures and reluctance in referring to the guidelines were identified as important contributory factors in East Africa [15]. An E-IMCI package has been tested in rural Tanzania and found to be feasible, to increase adherence to the IMCI protocol [16] and to improve health care delivery [17]. Motivation supported by all stakeholders seeking to improve implementation of IMCI could be instituted, as motivation was directly correlated to the quality and contribution of community volunteers to the implementation of community IMCI in Benin [18]. Supportive supervision and health system strengthening have been identified as key to the sustainable implementation of IMCI in Tanzania [19]. To address the barriers of ambiguous roles and responsibilities among stakeholders, which in some cases translates into lack of supply and logistics, a joint assessment of the situation by all stakeholders and streamlining IMCI implementation within the district through sound planning, training supervision and logistic support, was suggested by a study in rural Pakistan [20]. Similar recommendations are made in another study on integrated management of childhood and neonatal illness in India (IMNCI) [21]. The level of commitment of the health worker or the unit of the Ghana Health Service/Ministry of Health responsible for IMCI remains a key factor in its proper implementation.

The findings of this study suggest that a range of health workers perceive that the NHIS, which aims at health care financing for all Ghanaians, is gradually determining diagnosing, prescription and treatment of all illnesses, including malaria through government health facilities in the study health facilities. This is due to the strict application of its national guidelines [22] which defines what drugs will be paid and whether this is dependent upon a pathological diagnosis having taken place. Cost of any treatment not included in the guidelines will not be honoured by the NHIS. This means that the diagnosing, prescribing and treatment practice of some health workers and health facilities are primarily guided by NHIS funding rules.

Attendance to health facilities at both in- and outpatients departments was perceived to have increased due to the introduction of the NHIS, leading to increased workload and consequently the inability of care providers to strictly adhere to the revised IMCI guidelines. This perception is upheld by a study in southern Ghana [23] which showed an increase in both in- and outpatients in health facilities, attributable to the introduction of the NHIS. Removal of out-of-pocket payment has been shown to impact on health care-seeking behaviour in Ghana [24] but not on the health outcomes measured [24]. Factors accounting for this may be attributed to the broad benefits package that the scheme offers related to its new payment system and the growing membership.

Erratic supply of malaria RDTs and the use of the syndromic approach when facilities experience stock-outs of RDTs contribute to the perceptions and practice of malaria over-diagnosis. This may lead to inappropriate case management of malaria resulting in an increase in the economic burden to governments [25]. Findings from this study demonstrate the presence of erratic supply of malaria RDTs among facilities. Stock-outs of RDTs are common in other parts of Africa [26], where this impacts on fever case management and is a threat to the practice of test-based management of malaria.

Lack of trust of negative RDT results and the practice of prescribing an ACT despite this, was reported by the majority of health workers interviewed in this study. Similar findings have been reported from several other sites, including Zambia [27], Ghana and the Republic of Benin [28]. The Zambia study investigators suggested innovative approaches to health worker adherence to malaria diagnosis and treatment guidelines. Research findings by a study in Tanzania [29] identified the practice of prescribing an ACT even though a RDT result was negative as a solution to conflict in health worker-patient interaction in test-based management of malaria; formative research to understand malaria over-diagnosis is recommended. Lack of trust in RDT negatives has also been recorded among community health workers in community case management of malaria in some countries in sub-Saharan Africa [30]. Clinicians who received enhanced training in Cameroon on designing and implementing interventions to change clinicians’ practice in the management of uncomplicated malaria however strongly agreed that it is not appropriate to prescribe anti-malarials to a patient if they have a negative RDT result [31].

Preferences for either RDT or microscopy for malaria diagnosis varied. The comparability of results of both microcopy and some RDT kits [32-34] has been assessed and RDTs have been found to be acceptable to caregivers in rural Ghana [4], communities in Uganda [35] and among health workers in Uganda. The sensitivity and specificity of some RDTs have been well documented [36] and their specificity, sensitivity and acceptability as an alternative to microcopy established in a study in India [37]. RDTs have been found to improve malaria diagnosis in low level health care facilities in Uganda [38]. However, seasonality has been found to have some effect on the accuracy of RDTs as noted in a study in Burkina Faso in West Africa; different ranges of figures for RDT sensitivity and specificity were recorded in both dry and wet seasons [39]. In some parts of Uganda and Kenya, RDTs were found to be effective when used in low endemicity situations, but high false positive error rates occurred in areas with moderately high transmission [40]. It is worth noting that malaria microscopy is challenging: a study on the standardization of malaria microscopy in health facilities in Ethiopia on the availability of laboratory logistics and technical practices, concluded that most facilities fell below WHO standard [41]. Gaps were also noted in the continuity of supply of reagents and laboratory supplies for malaria microscopy in some health facilities in Ethiopia [42]. Perhaps in the future, modern methods, such as computer-based screening and visualization of blood smears may be a considered for malaria diagnostics [43]. The adoption of these methods by front-line health workers however may be problematic.

To be able to surmount the problem of lack of prompt supply of good quality malaria RDTs, the application of short message service (SMS) to deliver messages on RDT stock availability could be adopted as used in a pilot study ‘SMS for Life’ in Tanzania [44]. This system allows the use of mobile phones to send SMS messages on stock levels of drugs to supply points at different levels of the health system This has been found to be feasible for monitoring of stock levels of anti-malarials in Tanzania [45]. This method could be adopted and adapted to aid reporting of stock-outs of required supplies and to trigger the supply of such supplies, including the supply of RDTs and ACT.

Adherence to the revised guidelines may improve if revised to a shorter version. Training and continuous training of health workers on the new revised IMCI guidelines should be part of the curricula of educational institutions that train middle-level manpower for the Ministry of Health. Re-orientation of medical practitioners and other senior health officers involved in clinical consultation at the health centres and hospitals may be institutionalized by the various DHMTs as part of their quarterly activities.

## Conclusion

Implementation of the WHO-revised IMCI guidelines is confronted with a myriad of health system challenges. The perceptions of front-line health workers on the accuracy and need for RDTs together with the capacity of health systems to support implementation plays a crucial role. The NHIS guidelines on financing of diagnostics and treatments are influencing clinical decision-making in this setting. Further study is needed to understand the impact of the NHIS on the feasibility of integrating test-based management for malaria of the IMCI guidelines.
